# Neurological fallout of hyperglycemia: A case report of a rare presentation of osmotic demyelination syndrome

**DOI:** 10.5339/qmj.2026.19

**Published:** 2026-03-31

**Authors:** Rekha Gupta, Chandrika Sachar, Parul Kaktyan

**Affiliations:** 1 Department of Radiodiagnosis & Imaging, Government Medical College and Hospital, Chandigarh, India *Correspondance: Chandrika Sachar chandrikasachar@gmail.com

**Keywords:** Osmotic demyelination syndrome, hyperosmolar hyperglycemic state, diabetes mellitus, central pontine myelinolysis, MRI

## Abstract

**Background:**

: Osmotic demyelination syndrome (ODS) is a rare but potentially life-threatening neurological disorder. It is characterized by non-inflammatory demyelination, usually triggered by sudden shifts in serum osmolality. However, emerging evidence suggests that abrupt osmotic shifts caused by severe hyperglycemia, even in the absence of sodium imbalance, can also precipitate ODS.

**Case presentation:**

: A 57-year-old male with poorly controlled type 2 diabetes mellitus and chronic alcohol use presented with recurrent seizures and altered sensorium. Initial blood work revealed severe hyperglycemia (390 mg/dL), an elevated HbA1c level (15.2%), and a normal corrected serum sodium level (135 mEq/L). During hospitalization, the patient developed progressive neurological deterioration, culminating in quadriplegia.

**Imaging findings:**

: Initial non-contrast computed tomography (NCCT) of the brain was unremarkable. Magnetic resonance imaging (MRI) on day 11 showed symmetric T2/FLAIR (Fluid-Attenuated Inversion Recovery) hyperintensities in the central pons extending into the middle cerebellar peduncles. These findings, in the absence of diffusion restriction or enhancement, were characteristic of central pontine myelinolysis (CPM). No extrapontine involvement was observed.

**Differential diagnosis:**

: Radiological differentials considered included acute disseminated encephalomyelitis (ADEM), multiple sclerosis (MS), ischemic infarction, vasculitis, and brainstem glioma. However, the symmetric pontine involvement without enhancement or mass effect, along with the clinical context of hyperosmolar hyperglycemia, favored a diagnosis of ODS.

**Management and outcome:**

: Supportive therapy involving careful glucose correction, intravenous hydration, and close monitoring of metabolic parameters was initiated. The patient showed gradual neurological recovery during the hospital course.

**Conclusion:**

: This case highlights a rare presentation of CPM secondary to hyperosmolar hyperglycemia in the absence of hyponatremia. It underscores the importance of early recognition, neuroimaging, and cautious metabolic correction in diabetic patients presenting with new-onset neurological deficits.

## 1. INTRODUCTION

Osmotic demyelination syndrome (ODS) is a rare, life-threatening neurological disorder characterized by non-inflammatory demyelination in the central nervous system. This condition arises from oligodendrocyte apoptosis and the infiltration of macrophages, which break down myelin, typically following rapid changes in serum osmolality. ODS is also classified into central pontine myelinolysis (CPM), which affects the central pons, and extrapontine myelinolysis (EPM), which involves other brain regions.^1,2^

ODS is an infrequently encountered condition, with studies indicating a prevalence of approximately 0.06% among hospitalized patients.^3^

Historically, ODS has been strongly associated with the rapid correction of chronic hyponatremia. However, current understanding emphasizes that the key pathogenic mechanism involves a sudden osmotic imbalance, regardless of whether it arises from sodium, glucose, or other solutes. Clinically, ODS presents with delayed neurological symptoms such as altered mental status, quadriparesis, bulbar palsy, and respiratory compromise, often occurring within a week of osmotic correction.

While ODS is well documented in contexts such as chronic alcoholism, liver transplantation, and electrolyte disturbances,^1^ its occurrence in diabetes mellitus, particularly during or after a hyperosmolar hyperglycemic state (HHS), is rarely reported. Additional recognized risk factors for ODS include malnutrition, chronic alcohol use, hypokalemia, diuretic therapy, and aggressive fluid replacement.^4^ The pathophysiology in such cases likely mirrors that of classic ODS, driven by rapid shifts in serum osmolality.^5^

Maintaining osmotic balance is crucial for cell viability. In a patient with poorly controlled diabetes, persistent hyperglycemia likely induces osmotic stress, causing protein damage and aggregation in oligodendrocytes and contributing to demyelination. Hyperosmolarity may also promote the release of nitric oxide and other inflammatory mediators, disrupting tight junctions, while toxic substances released from injured endothelial cells could further damage oligodendrocytes, creating the pathogenic conditions for CPM.^6,7^

Diagnosing ODS in the context of severe hyperglycemia can be particularly challenging because its clinical manifestations closely resemble those of other neurological and metabolic complications of diabetes, including metabolic encephalopathy, stroke, infection, or cerebral edema. The delayed evolution of symptoms, along with the possibility of subtle or even normal early magnetic resonance imaging (MRI) findings, may further obscure timely recognition, underscoring the importance of heightened clinical suspicion in hyperosmolar states.

Although ODS is well established in association with the rapid correction of chronic hyponatremia, its development solely due to uncontrolled hyperglycemia remains exceedingly rare. Consequently, the diagnostic approach, underlying mechanisms, and clinical implications in such cases are not well delineated in current literature. This case helps bridge this knowledge gap by documenting a rare presentation of CPM precipitated purely by hyperosmolar hyperglycemia, thereby expanding the understanding of non-sodium-related triggers of osmotic demyelination.

This report highlights the link between uncontrolled hyperglycemia and ODS, underscoring the need for vigilance in managing patients with severe hyperglycemia.

## 2. CASE PRESENTATION

### 2.1. Clinical presentation

A 57-year-old male with a history of poorly controlled type 2 diabetes mellitus and chronic alcohol use presented to the emergency department with new-onset generalized tonic–clonic seizures and altered mental status. On the day of admission, he experienced five consecutive seizures, each accompanied by upward eye rolling, frothing at the mouth, and urinary incontinence. Postictally, the patient regained consciousness within 10–15 min, though he remained drowsy for approximately 30 min. His diabetes had been poorly managed for several years, with irregular use of insulin and metformin. There was no history of documented microvascular or macrovascular complications.

On general examination, the patient appeared clinically weak and fatigued. He was hemodynamically stable, with a blood pressure of 100/62 mmHg, a heart rate of 82 beats per minute, a respiratory rate of 20 breaths per minute, a body temperature of 36.5°C, and an oxygen saturation of 96% on room air. He had an average build, with a body mass index of 28. Neurologically, he was alert, with a Glasgow Coma Scale score of 15 (E4V5M6).

Cranial nerve examination was unremarkable, with no focal neurological deficits noted. Muscle bulk, tone, and power were preserved across all limbs, with strength graded at 4+/5. Sensory examination was normal, and there were no signs of cerebellar dysfunction. Deep tendon reflexes were brisk in all extremities, and bilateral plantar reflexes were extensor. Fundoscopic evaluation showed no evidence of papilledema or diabetic retinopathy. The patient had no prior history of electrolyte imbalances, correction of hyponatremia, liver transplantation, or malnutrition.

### 2.2. Investigations

On admission, his blood glucose was markedly elevated at 390 mg/dL, with an HbA1c of 15.2%, consistent with chronic poor glycemic control. Laboratory investigations (detailed in Table 1) also revealed hypoalbuminemia (serum albumin: 3.3 g/dL) and a corrected serum sodium level of 135 mEq/L. Seizure activity persisted during the hospital stay. The patient also had a known history of pulmonary tuberculosis and was currently receiving antitubercular therapy (ATT).

### 2.3. Imaging findings

**Figure 1:**
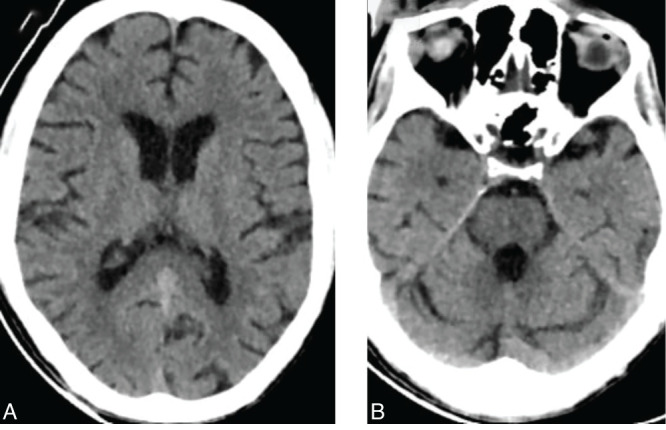
Axial (A and B) non-contrast CT images of the head reveal no evidence of abnormalities in the cerebral white matter, basal ganglia, or pons.

Upon admission, a non-contrast computed tomography (NCCT) scan of the brain (Figure 1) was performed, revealing no significant abnormalities in the brain parenchyma.

During the hospital stay, the patient was treated with intravenous insulin, intravenous levetiracetam, metformin, and ATT.

On the 11th day of hospitalization, he developed new focal neurological deficits, initially manifesting as left-sided weakness that progressed to quadriparesis, prompting further evaluation. These developments marked a significant clinical deterioration and prompted further neurological evaluation to determine the underlying cause.

MRI of the brain was performed on a 1.5 T MRI scanner (Achieva, Philips Medical Systems) using sequences including FLAIR (axial), T2-weighted imaging (axial and sagittal), 3D T1 (axial, coronal, and sagittal), and DWI (Diffusion-Weighted Imaging) / ADC (Apparent Diffusion Coefficient) images.

**Figure 2:**
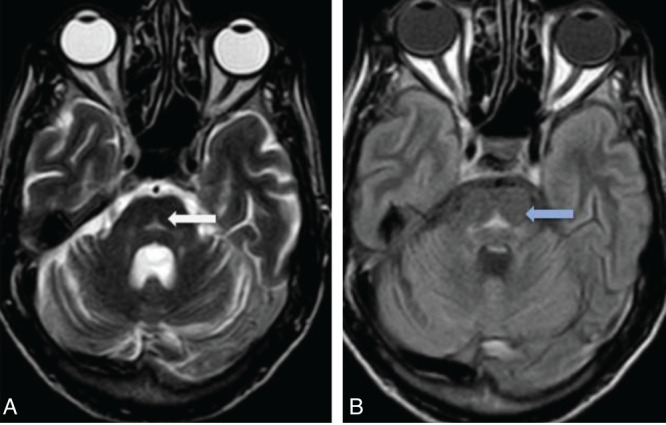
Axial T2-weighted (A) and FLAIR (B) images demonstrate a homogeneous, well-defined, triangular-shaped symmetric hyperintensity in the central pons (white arrow), without any associated mass effect. This hyperintense signal is also seen extending into the middle cerebellar peduncles (blue arrow in image B).

**Figure 3:**
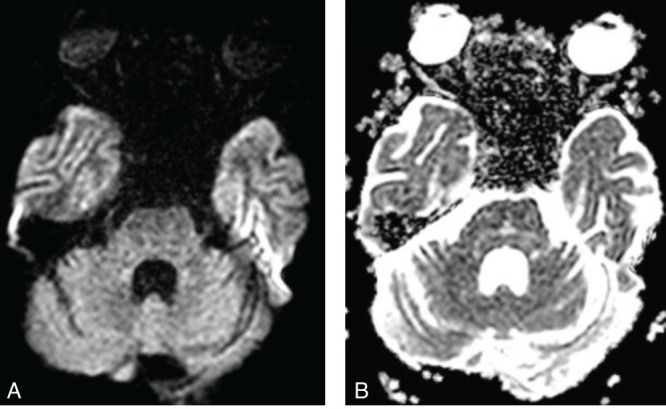
Axial DWI (A) and corresponding ADC (B) images showing no high signal intensity on the DWI image, with corresponding high signal on the ADC map.

**Figure 4:**
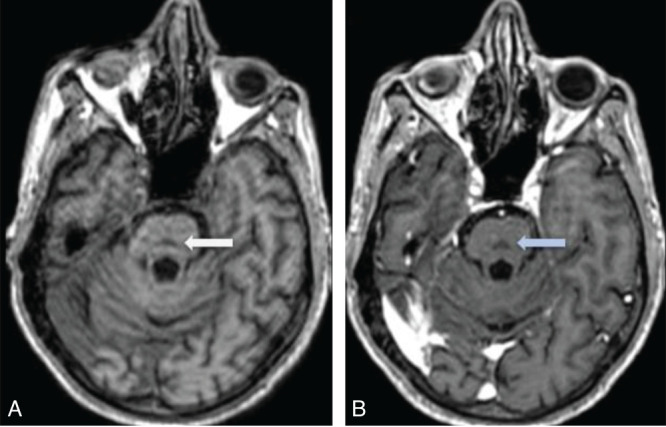
Axial T1 (A) and post-contrast axial T1 (B) images show hypointensity on T1-weighted images, with no contrast enhancement on post-gadolinium images (blue arrow).

**Figure 5:**
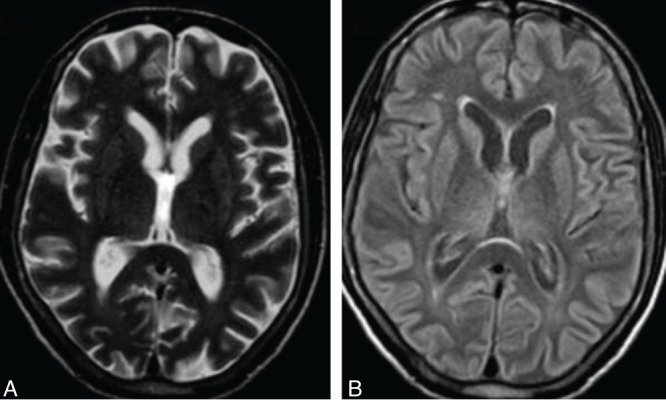
Axial T2 (A) and FLAIR (B) images show no abnormal T2/FLAIR hyperintensities in the basal ganglia, thalami, and both cerebral hemispheres, indicating no extrapontine abnormality on the present scan.

**Figure 6:**
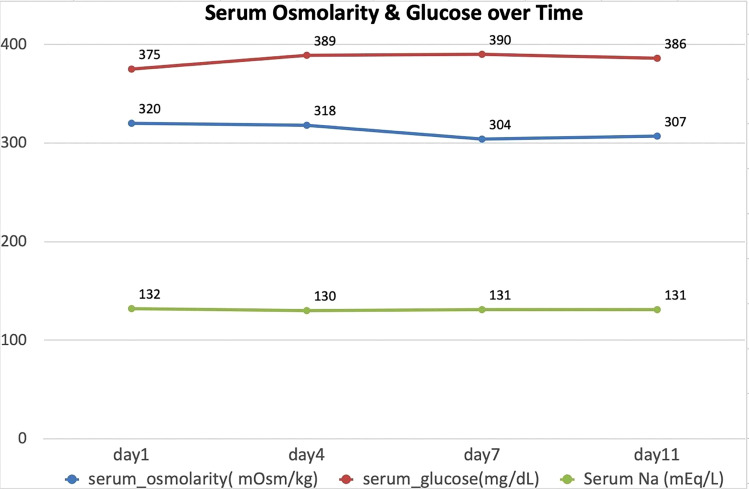
Time-series graph showing trends in serum osmolality, sodium, and glucose levels during hospitalization.

T2/FLAIR hyperintensities are observed in the central pons (Figure 2), appearing triangular and extending to the cerebellar peduncles, primarily the middle cerebellar peduncles. No diffusion restriction was seen in the central pons on DWI/ADC images (Figure 3). After administration of gadolinium, there was no enhancement of the pontine abnormality (Figure 4). The basal ganglia, thalami, and both cerebral hemispheres were within normal limits, without any extrapontine abnormalities (Figure 5). A final diagnosis of ODS with CPM secondary to hyperosmolar hyperglycemia was made. A time-series graph (Figure 6) shows trends of serum osmolality, sodium, and glucose levels during hospitalization.

### 2.4. Treatment received

Management focused on intravenous fluid resuscitation and insulin infusion to control the severely elevated blood glucose levels, with continuous monitoring to ensure metabolic stability.

Supportive measures, including gradual glucose correction, intravenous hydration, and close metabolic monitoring, resulted in progressive neurological improvement in the patient.

### 2.5. Outcome

In our patient, progressive neurological recovery and favorable long-term outcomes were observed following comprehensive supportive management and targeted rehabilitative therapy. Over several weeks to months, motor function improved substantially, with partial restoration of limb strength from the initial quadriplegia.

## 3. DISCUSSION

ODS is an uncommon yet potentially fatal neurological condition resulting from injury to the myelin sheath of brain cells. It is most commonly associated with the rapid correction of severe hyponatremia. The condition was first described by Adams et al. in 1959 in patients with alcoholism or malnutrition.^8^

ODS can present as CPM, extrapontine myelinolysis, or a combination of the two. Although its exact mechanism is not fully understood, several precipitating factors have been identified (in the absence of a recognized osmotic shift), including the rapid correction of longstanding hyponatremia, chronic alcohol abuse, liver disease, poor nutritional status, organ transplantation, hemodialysis, and extensive burn injuries.^9^

Rapid correction of hyponatremia can lead to a sudden rise in extracellular osmolality, overwhelming the brain’s adaptive mechanisms. In chronic hyponatremia, cerebral cells adjust by accumulating osmolytes to maintain osmotic balance. However, when sodium levels are restored too quickly, these cells become dehydrated and damaged due to their inability to readapt promptly. Oligodendrocytes, which are responsible for forming and maintaining myelin, are especially vulnerable, resulting in demyelination and neurological dysfunction.^10^

In ODS, demyelination predominantly affects region-specific oligodendrocytes, particularly those in the pons, basal ganglia, mesencephalon, and deep cortical layers. Conversely, glial cells in brain areas typically resistant to ODS remain unaffected by osmotic fluctuations.^2^ The characteristic focal involvement seen in CPM is thought to arise from the intricate grid-like architecture of intersecting and descending fiber tracts in the pons, possibly due to its inherent vascular vulnerability. The transverse pontocerebellar fibers are typically the first and most prominently affected, followed by the longitudinal rostrocaudal tracts. A key distinguishing feature from pontine infarction is the relative preservation of neurons and axons, highlighting the non-ischemic nature of the demyelinating process.^11^

In patients with CPM secondary to complicated diabetes mellitus, a broad array of neurological deficits may be observed. Common clinical features include dysphagia, dysarthria, and varying degrees of motor weakness, ranging from hemiplegia to paraparesis or quadriparesis. These motor deficits are often accompanied by reduced deep tendon reflexes, ataxia, and altered mental status. Cognitive disturbances, such as confusion, along with behavioral manifestations like agitation and disorientation, are also frequently observed.^12^

Computed tomography (CT) often lacks the sensitivity to detect the full extent of ODS; in contrast, MRI reliably reveals the classic demyelinating changes, making it the modality of choice for diagnosis. MRI typically demonstrates T1 hypointensity and T2/FLAIR hyperintensity, predominantly within the pons. A characteristic feature of ODS is the “trident sign”, marked by a symmetrical high signal in the central pons on T2/FLAIR images. T1 hypointense, T2 hyperintense, and FLAIR hyperintense signal alterations may also be observed in extrapontine regions, such as the basal ganglia, thalamus, cerebellum, hippocampus, and cerebral cortex.^13^

Earlier theories proposed that hyperglycemia-induced hyperosmolality disrupted the blood–brain barrier via osmotic shifts, causing plasma leakage, cerebral edema, and demyelination. More recent evidence suggests a multifactorial process, in which osmotic stress from fluctuating serum osmolality induces the release of nitric oxide, activates inflammatory cytokines, and promotes the production of toxic metabolites—factors that directly damage oligodendrocytes, leading to demyelination.^6,7^

ODS has been documented in diabetic patients, most frequently linked to HHS. In these cases, rapid reduction of blood glucose, repeated swings between hyperglycemia and hypoglycemia, or persistent hypernatremia have been implicated–each contributing to abrupt serum osmolality shifts that trigger demyelination.^5,14^ Notably, ODS has also occurred in diabetic individuals without HHS. In such cases, mechanisms include sudden insulin withdrawal leading to uncontrolled hyperglycemia or overly rapid correction of glucose levels. Both scenarios create osmotic disequilibrium, which injures oligodendrocytes and leads to demyelination.^6,15,16^

CPM in our patient is likely related to abrupt osmotic shifts affecting oligodendrocytes, leading to demyelination. Two primary mechanisms may explain this process in the context of hyperglycemia:

(i) Local inflammatory demyelination secondary to blood–brain barrier disruption: In the setting of chronically uncontrolled diabetes, osmotic stress can induce protein damage and aggregation within oligodendrocytes, contributing to demyelination. Hyperosmolarity may also trigger the release of nitric oxide and other inflammatory mediators, disrupting tight junctions, while toxic substances released from damaged endothelial cells can further injure oligodendrocytes.
(ii) Oligodendrocyte apoptosis induced by hypertonic stress: Rapid osmotic shifts from hyperglycemia can overwhelm adaptive cellular mechanisms, leading to apoptosis of oligodendrocytes and further myelin loss.^6,14^

CPM has been increasingly recognized in association with hyperosmolar hyperglycemia, even when serum sodium levels remain within the normal range. Burns et al.^5^ reported an elderly patient who developed CPM despite a normal serum sodium level, supporting the concept that rapid osmotic fluctuations from hyperglycemia alone can precipitate demyelination. Similarly, Yadav et al.^3^ described a case of complicated diabetes mellitus presenting with seizures and altered sensorium, underscoring that hyperglycemia-induced osmotic stress may act as an independent trigger for CPM, alongside other systemic conditions. More recently, Qu et al.^17^ documented a middle-aged patient with hyperglycemia-related CPM demonstrating characteristic pontine MRI findings, further confirming hyperosmolarity secondary to hyperglycemia as a direct pathogenic mechanism. Ramineni et al.^18^ described ODS in a patient with de novo type 2 diabetes mellitus presenting with a HHS, demonstrating that ODS can develop independently of sodium imbalance or its rapid correction.

Incorporating these references into the Discussion underscores both the novelty and scientific grounding of our case. By demonstrating CPM arising solely from hyperglycemia, with detailed radiological correlation and favorable clinical recovery after gradual glucose correction, our report reinforces hyperosmolar hyperglycemia as an independent pathogenic mechanism and highlights the clinical and radiological relevance of such rare presentations.

The radiologic differential diagnosis for ODS includes several mimics. Acute disseminated encephalomyelitis (ADEM) and multiple sclerosis (MS) may show T2/FLAIR hyperintensities but typically present with asymmetrical lesions, gadolinium enhancement, and a subacute or relapsing clinical course.

ADEM is an immune-mediated demyelinating disorder that typically presents with multifocal, asymmetric lesions involving both supratentorial and infratentorial white matter. Affected regions include the basal ganglia, thalami, and occasionally the spinal cord, while brainstem involvement is less frequent. The lesions are generally bilateral but asymmetric in distribution. Post-contrast imaging may reveal patchy or ring-like enhancement, reflecting the inflammatory nature of the disease.^19^

MS is the most common inflammatory demyelinating disease of the central nervous system. On MRI, it typically presents with T2 hyperintense lesions in at least two of the following four characteristic locations: juxtacortical or intracortical, periventricular, infratentorial, and spinal cord. Diagnosis is based on the principle of dissemination in time and space. Active lesions may show contrast enhancement, often with an “open ring” pattern, which is suggestive of ongoing inflammation.^20^

Vasculitis and ischemic infarction can also produce pontine signal changes; however, these usually involve the cerebral white matter and are rarely confined to the central pons. Brainstem gliomas, although possible, are generally associated with a mass effect and a more gradual clinical progression, making them unlikely in a sudden-onset presentation without visible expansion.^21^

In our patient, severe hyperglycemia was the predominant pathological driver of ODS, creating abrupt osmotic shifts that led to oligodendrocyte injury and central pontine demyelination. The patient’s history of chronic alcohol use may have modestly increased susceptibility, as alcohol can subtly influence sodium and water regulation through antidiuretic hormone (ADH) suppression and can independently contribute to myelin damage.^22^ Osmotic disturbances can occasionally arise during periods of heavy drinking or alcohol withdrawal due to reduced fluid and nutrient intake.^23^ However, in this case, laboratory and clinical findings strongly indicate that hyperglycemia was the principal factor, with chronic alcohol exposure serving only as a minor, permissive contributor rather than a primary cause.

ODS lacks a definitive treatment, and management primarily involves supportive care and addressing any underlying metabolic disturbances. In cases of hyperglycemia-induced ODS, it is imperative to correct serum glucose levels gradually, mirroring the cautious approach used for correcting hypernatremia. Continuous monitoring of neurological status, serum glucose concentrations, and serum osmolality is essential. Importantly, despite severe neurological deficits, evidence suggests that meaningful recovery from ODS is achievable in many cases.^9^

## 4. CONCLUSION

This case highlights the rare occurrence of CPM, a subtype of ODS, in the context of uncontrolled diabetes mellitus without overt hyponatremia. It underscores the importance of recognizing HHSs as potential triggers for ODS, even in the absence of rapid sodium correction. Heightened clinical awareness is essential to prevent, identify, and manage this potentially reversible yet life-threatening complication.

## ETHICAL APPROVAL

Informed consent for participation and publication of medical details was obtained from the patient. The confidentiality of patient data was ensured at all stages. The authors declare that ethics committee approval was not required for this case report.

## COMPETING INTERESTS

The authors have no conflicts of interest to declare.

## DATA AVAILABILITY STATEMENT

Data sharing is not applicable to this article, as no datasets were generated or analyzed during the current study.

## AUTHOR CONTRIBUTIONS STATEMENT

All authors contributed to Conceptualization, Methodology, Data Curation, Software & Validation, Investigation & Analysis, Visualization, Writing – Original Draft, Writing – Review & Editing, Resources, Supervision, Project Administration, and Funding Acquisition, and have approved the final manuscript.

## Figures and Tables

**Table 1:** Laboratory investigations during hospitalization, with reference ranges.

Test name	Results	Reference range
Albumin	3.3 g/dL	3.8–5.5 g/dL
Glycosylated HB (HBAIC)	15.2%	4.8–5.9%
Estimated average glucose (eAG)	390 mg/dL	
Sodium	131 mEq/L	135–145 mEq/L
Corrected sodium	135.6 mEq/L	135–145 mEq/L
Chloride	94 mEq/L	98–107 mEq/L
Glucose (fasting)	375 mg/dL	60.00–110.00 mg/dL
Glucose (post prandial)	683 mg/dL	0.00–140.00 mg/dL
Hb	10.9 gm%	12.00–18.00 gm%
Platelet count	106×10^9^/L	150.00–400.00×10^9^/L
Red cell indices (MCHC)	29.9%	30.00–36.00%
Serum osmolality	320 mOsm/kg H_2_O	275–295 mOsm/kg H_2_O
Urea	24 mg/dL	15–45 mg/dL
Creatinine	0.7 mg/dL	0.8–1.8 mg/dL
CRP (quantitative)	14 mg/L	0–5 mg/L
Calcium	8.2 mg/dL	8–10.4 mg/dL
